# High Prevalence of Multidrug-Resistant *Klebsiella pneumoniae* Harboring Several Virulence and β-Lactamase Encoding Genes in a Brazilian Intensive Care Unit

**DOI:** 10.3389/fmicb.2018.03198

**Published:** 2019-01-22

**Authors:** Roumayne L. Ferreira, Brenda C. M. da Silva, Graziela S. Rezende, Rafael Nakamura-Silva, André Pitondo-Silva, Emeline Boni Campanini, Márcia C. A. Brito, Eulália M. L. da Silva, Caio César de Melo Freire, Anderson F. da Cunha, Maria-Cristina da Silva Pranchevicius

**Affiliations:** ^1^Departamento de Genética e Evolução, Universidade Federal de São Carlos, São Carlos, Brazil; ^2^Laboratório Central de Saúde Pública do Tocantins, Palmas, Brazil; ^3^School of Dentistry, University of Ribeirão Preto, Ribeirão Preto, Brazil; ^4^Department of Cell Cycle and Cancer Biology, Oklahoma Medical Research Foundation, Oklahoma City, OK, United States

**Keywords:** *Klebsiella pneumoniae*, intensive care units, multi-drug resistance, β-lactams gene, virulence genes

## Abstract

*Klebsiella pneumoniae* is an important opportunistic pathogen that commonly causes nosocomial infections and contributes to substantial morbidity and mortality. We sought to investigate the antibiotic resistance profile, pathogenic potential and the clonal relationships between *K. pneumoniae* (*n* = 25) isolated from patients and sources at a tertiary care hospital’s intensive care units (ICUs) in the northern region of Brazil. Most of *K. pneumoniae* isolates (*n* = 21, 84%) were classified as multidrug resistant (MDR) with high-level resistance to β-lactams, aminoglycosides, quinolones, tigecycline, and colistin. All the 25 isolates presented extended-spectrum beta-lactamase-producing (ESBL), including carbapenemase producers, and carried the *bla*_KPC_ (100%), *bla*_TEM_ (100%), *bla*_SHV_ variants (*n* = 24, 96%), *bla*_OXA-1_ group (*n* = 21, 84%) and *bla*_CTX-M-1_ group (*n* = 18, 72%) genes. The K2 serotype was found in 4% (*n* = 1) of the isolates, and the K1 was not detected. The virulence-associated genes found among the 25 isolates were *mrk*D (*n* = 24, 96%), *fim*H-1 (*n* = 22, 88%), *ent*B (100%), *iut*A (*n* = 10, 40%), *ybt*S (*n* = 15, 60%). The genes related with efflux pumps and outer membrane porins found were *AcrAB* (100%), *tol*C (*n* = 24, 96%), *mdt*K (*n* = 22, 88%), *Omp*K35 (*n* = 15, 60%), and *Omp*K36 (*n* = 7, 28%). ERIC-PCR was employed to determine the clonal relationship between the different isolated strains. The obtained ERIC-PCR patterns revealed that the similarity between isolates was above 70%. To determine the sequence types (STs) a multilocus sequence typing (MLST) assay was used. The results indicated the presence of high-risk international clones among the isolates. In our study, the wide variety of MDR *K. pneumoniae* harboring β-lactams and virulence genes strongly suggest a necessity for the implementation of effective strategies to prevent and control the spread of antibiotic resistant infections.

## Introduction

*Klebsiella pneumoniae* is a Gram-negative opportunistic bacterium that causes infections in hospitalized or otherwise immunocompromised individuals (Gorrie et al., 2017). Currently, *K. pneumoniae* is showing a high resistance to a broad spectrum of drugs including beta-lactam antibiotics, fluoroquinolones, and aminoglycosides (Fair and Tor, 2014; Dsouza et al., 2017). This resistance is resulting in a growing worldwide problem regarding the choice of effective antibiotic treatment for hospital-acquired infections (Davies and Davies, 2010).

Antibiotics of the β-lactam group are commonly prescribed worldwide and include penicillins, cephalosporins, monobactams, and carbapenems (Samaha-Kfoury and Araj, 2003; Ur Rahman et al., 2018). The production of β-lactamase enzymes by the presence of β-lactam-insensitive cell wall transpeptidases, or the active expulsion of β-lactam molecules from Gram-negative bacteria represent the main indications of β-lactam antibiotic resistance (Wilke et al., 2005). Carbapenems are the β-lactams of choice for the treatment of infections caused by extended-spectrum beta-lactamase (ESBL)-producing bacteria (Karuniawati et al., 2013; Okoche et al., 2015), such as *K. pneumoniae*. These antibiotics are also considered the last resort for the management of life-threatening health-care-associated infections (Amjad et al., 2011). Unfortunately, bacterial resistance to carbapenems has been increased and is well documented (Paterson and Bonomo, 2005; World Health Organization [WHO], 2014), and has also been further complicated by the production of β-lactamases, efflux pumps, and mutations that alter the expression and/or function of porins and penicillin-binding proteins (PBPs) (Papp-Wallace et al., 2011).

Antimicrobial resistance is commonly related to the spread of transmissible plasmids and the acquisition of resistance genes that normally occur by horizontal gene transfer, which may also carry virulence determinants (Derakhshan et al., 2016). For pathogen survival, the acquisition of resistance and virulent traits is necessary (Da Silva and Mendonça, 2012), and some reports suggest that such may have an essential role in the pathogenesis of *K. pneumoniae* infections (Vila et al., 2011). Capsule, lipopolysaccharide (LPS), fimbriae (types 1 and 3), and siderophores are virulence factors that contribute to the pathogenicity of *K. pneumoniae*. *K. pneumoniae* strains can synthesize capsules of any of the serotypes *K1* to *K78;* however, K1 and K2 can also be associated with increased pathogenicity (Paczosa and Mecsas, 2016).

Here, we show the antibiotic resistance profile, pathogenic potential, and clonal relationships among *K. pneumoniae* isolated from patients and sources at a tertiary care hospital’s intensive care units (ICUs) in the northern region of Brazil.

## Materials and Methods

### Bacterial Strains

Twenty-five *K. pneumoniae* clinical isolates were collected from patients and devices at a tertiary care hospital’s ICUs in the state of Tocantins, located in the northern region of Brazil, between January 2014 and May 2015. All *K. pneumoniae* were collected at the bed-side, and then transported to the microbiology laboratory immediately for inoculation on proper culture media and preliminary analysis. Thereafter, the bacterial cultures were sent to the Central Laboratory of Public Health of Tocantins (LACEN/TO), a reference unit from the Brazilian Ministry of Health that receives samples for surveillance of antimicrobial resistance and which is usually located in the capital city of each federal state of Brazil. Strains were isolated from the following sources: tracheal aspirate, rectal swab, surgical drain, wound, catheter tip, cerebrospinal fluid, abscess, urine, and sputum.

### Ethics Statement

In this work, all *K. pneumoniae* and the anonymous archival data related patient age, gender, and sample type were obtained from LACEN/TO (data’s owner). The study was approved by the Committee of Ethics in Human Research of the Federal University of São Carlos (no. 1.088.936). Permission to conduct the present study was obtained from the Health Department of the State of Tocantins (Secretaria da Sauìde do Estado do Tocantins – SESAU) and LACEN/TO. Patient consent was not required, since the data presented in this study do not relate to any specific person or persons.

### Phenotypic Detection of Antibiotic Resistance and Carbapenemase Productions

The identification of *K. pneumoniae* and the evaluation of their susceptibility profiles were performed using the VITEK 2 system (bioMérieux, Inc., Hazelwood, MO, United States) following the Clinical and Laboratory Standards Institute guidelines (Clinical and Laboratory Standards Institute [CLSI], 2017). All *K. pneumoniae* was tested for their resistance against the following 15 antibiotics: ampicillin/sulbactam (SAM), piperacillin/tazobactam (TZP), cefuroxime (CXM), cefoxitin (FOX), ceftazidime (CAZ), ceftriaxone (CRO), cefepime (FEP), ertapenem (ERP), imipenem (IMP), meropenem (MEM), amikacin (AMK), gentamicin (GEN), ciprofloxacin (CIP), tigecycline (TGC), and colistin (CST). Susceptibility to TGC was interpreted using breakpoints proposed by the European Committee on Antimicrobial Susceptibilities Testing (EUCAST)^1^.

Determination of the production of carbapenemase was carried out by modified Hodge test, synergy test, and the ethylenediaminetetraacetic acid (EDTA) test under the CLSI guidelines (Clinical and Laboratory Standards Institute [CLSI], 2017) and as described elsewhere (Miriagou et al., 2010; Nordmann et al., 2011; Okoche et al., 2015).

Multidrug-resistant (MDR) *K. pneumoniae* isolates were defined by non-susceptibility to at least one agent in three or more antibiotic categories (Magiorakos et al., 2012).

### Genomic DNA Extraction

Genomic DNA was extracted from an overnight culture using the Wizard^®^ Genomic DNA Purification Kit (Promega, Madison, WI, United States). The concentration of the DNA extract and purity was determined by measuring absorbance at wavelengths of 260 nm and 280 nm (NanoVue Plus; GE Healthcare Life Sciences, Marlborough, MA, United States). The integrity of genomic DNA was tested by way of electrophoresis.

### Detection of Multidrug Resistance Genes

The detection of resistance genes was performed by polymerase chain reaction (PCR) and their identities confirmed by sequencing. Isolates were screened by PCR amplification using specific primers for the detection of ESBL-encoding genes (*bla*_TEM_; *bla*_SHV_; *bla*_CTX-M_; and *bla*_OXA1,4,and30_), carbapenemases genes (*bla*_KPC_, *bla*_VIM_, *bla*_IMP_, *bla*_NDM_, and *bla*_OXA48_), a tetracycline resistance gene (*tetB*), and a CST resistance gene (*mcr-1*). Moreover, efflux pump (*AcrAB, mdtK*, and *ToIC*), and porin-coding (*Omp*K35 and *Omp*K36) genes were also investigated. The specific primers (Exxtend, São Paulo, Brazil) and the length of expected PCR products are presented in Table 1. Amplicons were analyzed by gel electrophoresis in 1.5% agarose and visualized under ultraviolet (UV) light. The forward primers were used for DNA sequencing.

**Table 1:** Sequences of primes used for detection of resistance genes and outer membrane porins.

Resistance targeted	Sequence (5’–3’), F/R	*T*_m_ (°C)	Amplicon size (bp)	Reference
*bla*_KPC_	CGTCTAGTTCTGCTGTCTTG CTTGTCATCCTTGTTAGGCG	61,3	797	Poirel et al., 2011
*bla*_TEM_	TGCGGTATTATCCCGTGTTG TCGTCGTTTGGTATGGCTTC	63	296	Xiong et al., 2007
*bla*_CTX-M-1group_, (including *bla*_CTX-M-1,3,__10,__11and12_)	ACAGCGATAACGTGGCGATG TCGCCCAATGCTTTACCCAG	64	216	Xiong et al., 2004
*bla*_SHV variants_	AGCCGCTTGAGCAAATTAAAC ATCCCGCAGATAAATCACCAC	55,6	712	Dallenne et al., 2010
*bla*_OXA-1,4and30_	GGCACCAGATTCAACTTTCAAG GACCCCAAGTTTCCTGTAAGTG	63	563	Dallenne et al., 2010
*bla*_OXA-48_	GCGTGGTTAAGGATGAACAC CATCAAGTTCAACCCAACCG	55	438	Poirel et al., 2011
*bla*_IMP_	CTACCGCAGCAGAGTCTTTGC ACAACCAGTTTTGCCTTACC	55	587	Martins et al., 2007
*bla*_VIM_	AAAGTTATGCCGCACTCACC TGCAACTTCATGTTATGCCG	55	865	Yan et al., 2001
*bla*_NDM_	GCAGCTTGTCGGCCATGCGGGC GGTCGCGAAGCTGAGCACCGCAT	60	782	Doyle et al., 2012
*gyr*A	TACCGTCATAGTTATCCACGA GTACTTTACGCCATGAACGT	61,3	387	Wiuff et al., 2000
*tet*B	CAGTGCTGTTGTTGTCATTAA GCTTGGAATACTGAGTGTAA	59,7	571	Call et al., 2003
*mcr*-1	CGGTCAGTCCGTTTGTTC CTTGGTCGGTCTGTAGGG	51,6	309	Liu et al., 2015
*Acr*AB	ATCAGCGGCCGGATTGGTAAA CGGGTTCGGGAAAATAGCGCG	58	312	Wasfi et al., 2016
*Tol*C	ATCAGCAACCCCGATCTGCGT CCGGTGACTTGACGCAGTCCT	61	525	Wasfi et al., 2016
*mdt*K	GCGCTTAACTTCAGCTCA GATGATAAATCCACACCAGAA	52	453	Wasfi et al., 2016
*Omp*K35	CTCCAGCTCTAACCGTAGCG GGTCTGTACGTAGCCGATGG	58	241	Wasfi et al., 2016
*Omp*K36	GAAATTTATAACAAAGACGGC GACGTTACGTCGTATACTACG	48	305	Wasfi et al., 2016


### Serotypes and Virulence-Associated Genes Detection

Polymerase chain reaction was used to detect the presence of capsule serotypes (K1 and K2), and virulence-associated genes. These virulence-associated genes included those encoding for regulators of mucoid phenotype A (*rmp*A), type 1 and type 3 adhesins (*fim*H-1 and *mrk*D), enterobactin (*ent*B), yersiniabactin (*Ybt*S), and aerobactin siderophore system (*iut*A). Isolated DNA samples were screened using specific primers (Exxtend, São Paulo, Brazil) for the detection of virulence genes (Table 2). The forward primers were used for DNA sequencing.

**Table 2:** Sequences of primers used for detection of virulence genes.

Gene	Primer sequence (5’–3’), F/R	Amplicon size (bp)	*T*_m_ (°C)	Reference
*rmp*A	ACTGGGCTACCTCTGCTTCA CTTGCATGAGCCATCTTTCA	535	54	Siu et al., 2011
*fim*H-1	TGCTGCTGGGCTGGTCGATG GGGAGGGTGACGGTGACATC	550	61	Schembri et al., 2005
*mrk*D	CCACCAACTATTCCCTCGAA ATGGAACCCACATCGACATT	226	54	El Fertas-Aissani et al., 2013
*iut*A	GGGAAAGGCTTCTCTGCCAT TTATTCGCCACCACGCTCTT	920	56	Compain et al., 2014
*ent*B	CTGCTGGGAAAAGCGATTGTC AAGGCGACTCAGGAGTGGCTT	385	57	Wasfi et al., 2016
*ybt*S	GACGGAAACAGCACGGTAAA GAGCATAATAAGGCGAAAGA	242	52	Compain et al., 2014
K1	GGTGCTCTTTACATCATTGC GCAATGGCCATTTGCGTTAG	1283	47	Fang et al., 2007
K2	GGATTATGACAGCCTCTCCT CGACTTGGTCCCAACAGTTT	908	45	Fang et al., 2007


### Sequence Analysis of Resistance and Virulence Genes

The PCR products were extracted from agarose gels, using the Illustra GFX PCR DNA and Gel Band Purification Kit (GE Healthcare, Chicago, IL, United States), and some of them were randomly selected for DNA Sanger sequencing (Macrogen Inc., Korea). The nucleotide sequences of the corresponding genes of the isolates were submitted to the GenBank database with accession numbers MK106173 to MK106187. The sequences were edited with Ugene v1.18.0 (Okonechnikov et al., 2012). Each sequence was compared using BlastN tools^2^ with the *K. pneumoniae* genome as the reference. Access to genetic heritage was approved by the National System for the Management of Genetic Heritage (SisGen) (no. AFF27ED).

### Enterobacterial Repetitive Intergenic Consensus Polymerase Chain Reaction

Enterobacterial repetitive intergenic consensus PCR (ERIC-PCR) analysis was performed to evaluate the genetic similarity among the bacterial isolates used in this study. ERIC-PCR reactions were executed as previously described by Versalovic et al. (1994), using the primers ERIC1R (5′-ATGTAAGCTCCTGGGGATTCAC-3′) and ERIC2 (5′-AAGTAAGTGACTGGGGTGAGCG-3′). All amplifications were carried out in a total volume of 50 μL, using the enzyme TaKaRa Ex Taq^®^ DNA Polymerase (Takara Bio, Kusatsu, Japan), while standardizing the amount of 100 ng of DNA template for each isolate. The amplified products were separated by 1.5% agarose gel electrophoresis and stained with ethidium bromide using UV radiation for visualization of the bands. The band profile analysis was performed using the BioNumerics program version 5.1 (Applied Maths, Keistraat, Belgium) for construction of the similarity dendrogram by the unweighted pair group mean method, Dice’s similarity coefficient, and 1% band position tolerance. Only bands representing amplicons between 300 bp and 3,000 bp were considered for this analysis. The ERIC-PCR assays were performed in triplicate.

### MLST

Ten isolates belonging to the main clusters of the dendrogram obtained by ERIC-PCR were selected for multilocus sequence typing (MLST). Information on the methodology used, including the primers and PCR reaction conditions, is available in the MLST database for *K. pneumoniae*^3^. The alleles and sequence types (STs) of each isolate studied by MLST were determined using the MLST database platform for *K. pneumoniae*.

The determination of the clonal and epidemiological relationships and the formation of clonal complexes (CCs), were completed by analyzing a genetic similarity diagram constructed with the aid of the eBURSTv3 program (eBURSTv3 has been developed and is hosted at The Department of Infectious Disease Epidemiology Imperial College London) (Feil et al., 2004).

### Statistical Analysis

The statistical analysis was performed using Fisher’s exact test (*p* ≤ 0.05).

## Results

### Antibiotic Resistance Patterns

In the present study, a total of 25 *K. pneumoniae* strains were isolated from samples collected from ICUs patients and devices of a tertiary hospital located in the northern region of Brazil. Most *K. pneumoniae* isolates were obtained from a rectal swab (56%; *n* = 14), followed by tracheal aspirate (16%, *n* = 4), urine (4%, *n* = 1), cerebrospinal fluid (4%, *n* = 1), wound (4%, *n* = 1), sputum (4%, *n* = 1), abscess (4%, *n* = 1), surgical drain (4%, *n* = 1), and catheter tip (4%, *n* = 1). A statistical difference was found only between the rectal swab and tracheal aspirate for isolates with resistance to the antibiotic TGC (Supplementary Table S1). Patients ages ranged from 1 day to 75 years (median age: 39 years old), and no significant differences were found regarding age group or gender and anti-microbial resistance. *K. pneumoniae* strains tested were resistant to all β-lactams (SAM, TZP, CXM-S, CXM, FOX, CAZ, CRO, FEP, ETP, IPM, MEM). These isolates also showed different degrees of resistance to other antibiotics like GEN (80%, *n* = 20), CIP (64%, *n* = 16), TGC (52%, *n* = 13) CST (36%, *n* = 9), and AMK (4%, *n* = 1). Demographic characteristics of the patients and antibiotic resistance profiles of the *K. pneumoniae* isolates to the 16 antibiotics tested are shown in Table 3.

**Table 3:** Characteristics of the patients and antibiotic resistance profile of the *K. pneumoniae.*

Characteristic	% (*n*)	Antibiotics	% (*n*) profile	
**Sex**		**Beta lactams**		
Female	44.0 (11)	(SAM, TZP, CXM-S, CXM, FOX, CAZ,	100.0 (25)	R
Male	56.0 (14)	CRO, FEP, ETP, IPM, MEM)		
**Age (years)**				
0–18	28.0 (7)	**Gentamycin**	80.0 (20)	R
19–59	36.0 (9)	(GEN)	20.0 (5)	S
60 or more	36.0 (9)			
**Sample type**		**Amikacin**	4.0 (1)	R
Tracheal aspirate	16.0 (4)	(AMK)	96.0 (24)	S
Rectal swab	56.0 (14)			
Drain	4.0 (1)	**Ciprofloxacin**	64.0 (16)	R
Wound	4.0 (1)	(CIP)	36.0 (9)	S
Catheter tip	4.0 (1)			
Cerebrospinal fluid	4.0 (1)		52.0 (13) 48.0 (12)	
Abscess	4.0 (1)	**Tigecycline**		R
Urine	4.0 (1)	(TGC)		S
Sputum	4.0 (1)			
		**Colistin**	36.0 (9)	R
		(CST)	64.0 (16)	S


*Antibiotics: SAM (ampicillin-sulbactam), TZP (piperacillin-tazobactam), CXM-S (cefuroxime sodium), CXM (cefuroxime axetil), FOX (cefoxitin), CAZ (ceftazidime), CRO (ceftriaxone), FEP (cefepime), ETP (ertapenem), IPM (imipenem), MEM (meropenem), GEN (gentamicin), AMK (amikacin), CIP (ciprofloxacin), TGC (tigecycline), CST (colistin). Profile: R, resistance rate; S, sensitivity rate; *n*, number.*

### Detection of Genes Coding for Outer Membrane Porins and Multidrug-Resistant Efflux Pumps and Antimicrobial Susceptibility

The majority of isolates (84%, 21/25) were classified as MDR with high-level resistance to at least one agent in three or more antibiotic categories. Among the MDR *K. pneumoniae*, all (100%, 21/21) isolates contained both *ArcA*B and *Tol*C efflux pumps genes; 86% (18/21) had *AcrAB, mdtK*, and *ToIC* genes, simultaneously; and only 14% (3/21) of isolates did not present with the *mdtK* multidrug efflux gene. PCR results showed that 33% (7/21) of isolates lacked both *Omp*K35 and *Omp*K36 porin genes, while 38% (8/21) of isolates lacked the *Omp*K36 gene.

Of the four isolates (Kp2, Kp67, Kp74, and Kp75) that did not show MDR profiles, three (Kp2, Kp74, and Kp75) had the *Acr*AB, *mdt*K and *ToI*C genes but not the *Omp*K35 and *Omp*K36 porin genes and one isolate (Kp67) carried both the *Acr*AB, and *mdtK* efflux pumps genes and the *Omp*K35 and *Omp*K36 porin genes. The antibiotic resistance profiles of the *K. pneumoniae* isolates are presented in Table 4. PCR amplification results for these genes are shown in Supplementary Figure S1.

**Table 4:** Antimicrobial resistance of *Klebsiella pneumoniae* isolates and presence of genes coding for outer membrane porins and efflux pumps.

*Isolate no.*	Antimicrobial resistance	MDR	Genes coding for porins and efflux pumps				
			
			*Omp*K*35*	*Omp*K36	*TolC*	*AcrA*B	*mdt*K
Kp1	sam, tzp, cxm, cxm-s, fox, caz, cro, fep, etp, ipm, mem, gen, cip, tgc	+	+	+	+	+	+
Kp2^∗^	sam, tzp, cxm, cxm-s, fox, caz, cro, fep, etp, ipm, mem, gen	-	-	-	+	+	+
Kp3	sam, tzp, cxm, cxm-s, fox, caz, cro, fep, etp, ipm, mem, amk, gen, cip, tgc, cst	+	+	-	+	+	+
Kp4	sam, tzp, cxm, cxm-s, fox, caz, cro, fep, etp, ipm, mem, gen, cst	+	-	-	+	+	+
Kp6	sam, tzp, cxm, cxm-s, fox, caz, cro, fep, etp, ipm, mem, gen, cip, tgc	+	+	-	+	+	+
Kp7	sam, tzp, cxm, cxm-s, fox, caz, cro, fep, etp, ipm, mem, gen, cip, tgc	+	+	+	+	+	+
Kp8	sam, tzp, cxm, cxm-s, fox, caz, cro, fep, etp, ipm, mem, cip, tgc	+	+	-	+	+	+
Kp16	sam, tzp, cxm, cxm-s, fox, caz, cro, fep, etp, ipm, mem, gen, cip, tgc	+	+	+	+	+	+
Kp17	sam, tzp, cxm, cxm-s, fox, caz, cro, fep, etp, ipm, mem, gen, cst	+	-	-	+	+	+
Kp21	sam, tzp, cxm, cxm-s, fox, caz, cro, fep, etp, ipm, mem, gen, cip	+	+	+	+	+	-
Kp25	sam, tzp, cxm, cxm-s, fox, caz, cro, fep, etp, ipm, mem, gen, cip, tgc, cst	+	-	-	+	+	+
Kp27	sam, tzp, cxm, cxm-s, fox, caz, cro, fep, etp, ipm, mem, cip, tgc, cst	+	+	-	+	+	+
Kp39	sam, tzp, cxm, cxm-s, fox, caz, cro, fep, etp, ipm, mem, gen, cip, tgc	+	+	-	+	+	+
Kp53	sam, tzp, cxm, cxm-s, fox, caz, cro, fep, etp, ipm, mem, gen, cip, tgc, cst	+	-	-	+	+	+
Kp60	sam, tzp, cxm, cxm-s, fox, caz, cro, fep, etp, ipm, mem, gen, cst	+	-	-	+	+	+
Kp62	sam, tzp, cxm, cxm-s, fox, caz, cro, fep, etp, ipm, mem, gen, cip, tgc	+	-	-	+	+	+
Kp66	sam, tzp, cxm, cxm-s, fox, caz, cro, fep, etp, ipm, mem, gen, cip	+	+	-	+	+	-
Kp67**^∗^**	sam, tzp, cxm, cxm-s, fox, caz, cro, fep, etp, ipm, mem	-	+	+	-	+	+
Kp68	sam, tzp, cxm, cxm-s, fox, caz, cro, fep, etp, ipm, mem, cip, tgc	+	+	+	+	+	+
Kp69	sam, tzp, cxm, cxm-s, fox, caz, cro, fep, etp, ipm, mem, gen, cst	+	-	-	+	+	+
Kp70	sam, tzp, cxm, cxm-s, fox, caz, cro, fep, etp, ipm, mem, cip, tgc	+	+	+	+	+	+
Kp73	sam, tzp, cxm, cxm-s, fox, caz, cro, fep, etp, ipm, mem, gen, cst	+	+	-	+	+	+
Kp74**^∗^**	sam, tzp, cxm, cxm-s, fox, caz, cro, fep, etp, ipm, mem, gen	-	-	-	+	+	+
Kp75**^∗^**	sam, tzp, cxm, cxm-s, fox, caz, cro, fep, etp, ipm, mem, gen	-	-	-	+	+	+
Kp77	sam, tzp, cxm, cxm-s, fox, caz, cro, fep, etp, ipm, mem, gen, cip	+	+	-	+	+	-


*Antibiotics. β-lactams: SAM (ampicillin-sulbactam), TZP (piperacillin-tazobactam), CXM-S (cefuroxime sodium), CXM (cefuroxime axetil), FOX (cefoxitin), CAZ (ceftazidime), CRO (ceftriaxone), FEP (cefepime), ETP (ertapenem), IPM (imipenem), MEM (meropenem); aminoglycosides: GEN (gentamicin) and AMK (amikacin); quinolones: CIP. (ciprofloxacin); glycylcycline: TGC (tigecycline) and polymyxin E: CST (colistin). MDR (multidrug-resistant) = resistance to at least one agent in three or more antibiotic categories. ^∗^Isolates that did not susceptible to at least three categories of antimicrobials.*

### Antibiotic Resistance and Virulence-Associated Genes Detection

The distributions of the antibiotic resistance gene and virulence factors are shown in Table 5. All the 25 isolates were positive for the *bla*_KPC_ gene. In addition, the *K. pneumoniae* isolates carried the *bla*_TEM_ (100%, *n* = 25), *bla*_SHV_ group (96%, *n* = 24), *bla*_OXA-1_ group (84%, *n* = 21), and *bla*_CTX-M-1_ group (72%, *n* = 18) ESBL-encoding genes. The *bla*_IMP_, *bla*_OXA-48_, *bla*_NDM_, *bla*_VIM,_ *mcr-1* and *tet*(B) genes were not detected. It was found that a high number of *bla*_SHV_ in this study that may be associated with the presence of *bla*_SHV -1_, which it is reported to be universal in *K. pneumoniae* infection (Babini and Livermore, 2000). Additional PCR amplification results are shown in Supplementary Figures S2, S3.

**Table 5:** Distribution of serotypes, resistance and virulence genes in *K. pneumoniae* strains.

Resistance genes		Virulence genes																					

		*bla_KPC_*	*bla_OXA_*	*bla_OXA-48_*	*bla_VIM_*	*bla_IMP_*	*bla_NDM_*	*bla_TEM_*	*bla_SHVvariants_*	*bla_CTX-M1group_*	*mcr-1*	*tetB*	*fimH-1*	*mrkD*	*entB*	*ybtS*	*iutA*	*RmpA*	*TolC*	*AcrAB*	*mdtK*	K1	K2
**Isolate**	**Sample type**																						
Kp1	Rectal swab	•	•					•	•	•			•	•	•				•	•	•		
Kp2	Rectal swab	•	•					•	•	•				•	•	•	•		•	•	•		
Kp3	Rectal swab	•	•					•	•				•	•	•				•	•	•		
Kp4	Urine	•	•					•	•	•			•		•	•			•	•	•		
Kp6	Rectal swab	•						•	•				•	•	•	•			•	•	•		
Kp7	Rectal swab	•	•					•	•	•			•	•	•				•	•	•		
Kp8	Rectal swab	•	•					•	•				•	•	•				•	•	•		
Kp16	Rectal swab	•	•					•	•	•			•	•	•				•	•	•		
Kp17	Rectal swab	•	•					•	•	•			•	•	•	•	•		•	•	•		
Kp21	Tracheal aspirate	•	•					•	•	•			•	•	•				•	•			
Kp25	Rectal swab	•	•					•	•	•			•	•	•	•	•		•	•	•		
Kp27	Rectal swab	•						•	•				•	•	•				•	•	•		•
Kp39	Rectal swab	•	•					•	•	•			•	•	•	•	•		•	•	•		
Kp53	Rectal swab	•	•					•	•	•			•	•	•	•	•		•	•	•		
Kp60	Cerebrospinal fluid	•	•					•	•	•			•	•	•	•	•		•	•	•		
Kp62	Drain	•	•					•	•	•			•	•	•	•	•		•	•	•		
Kp66	Catheter tip	•	•					•	•				•	•	•				•	•			
Kp67	Tracheal aspirate	•						•					•	•	•					•	•		
Kp68	Rectal swab	•	•					•	•				•	•	•	•	•		•	•	•		
Kp69	Wound	•	•					•	•	•			•	•	•	•	•		•	•	•		
Kp70	Sputum	•						•	•	•				•	•				•	•	•		
Kp73	Abscess	•	•					•	•	•			•	•	•				•	•	•		
Kp74	Rectal swab	•	•					•	•	•				•	•	•	•		•	•	•		
Kp75	Tracheal aspirate	•	•					•	•	•			•	•	•				•	•	•		
Kp77	Tracheal aspirate	•	•					•	•	•			•	•	•				•	•			
Genes present (%)		100	84	0	0	0	0	100	96	72	0	0	88	96	100	60	40	0	96	100	88	0	4


Polymerase chain reaction analysis demonstrated that the *fim*H-1 and *mrk*D genes, encoding type 1 and type 3 fimbrial adhesins, were present in 88% (22/25) and 96% (24/25) of isolates, respectively. Additionally, the enterobactin (*ent*B) gene was found in 100% (25/25), the yersiniabactin (*ybt*S) gene in 60% (15/25) and the aerobactin siderophore system (*iut*A) gene in 40% (10/25) of isolates. The regulators of the mucoid phenotype A (*rmp*A) gene were not detected. Only one isolate (4%), recovered from swab rectal, presented the capsular serotype K2, and the capsular K1 was not found (Table 5 and Supplementary Figure S1).

### Enterobacterial Repetitive Intergenic Consensus Polymerase Chain Reaction

**FIGURE 1:**
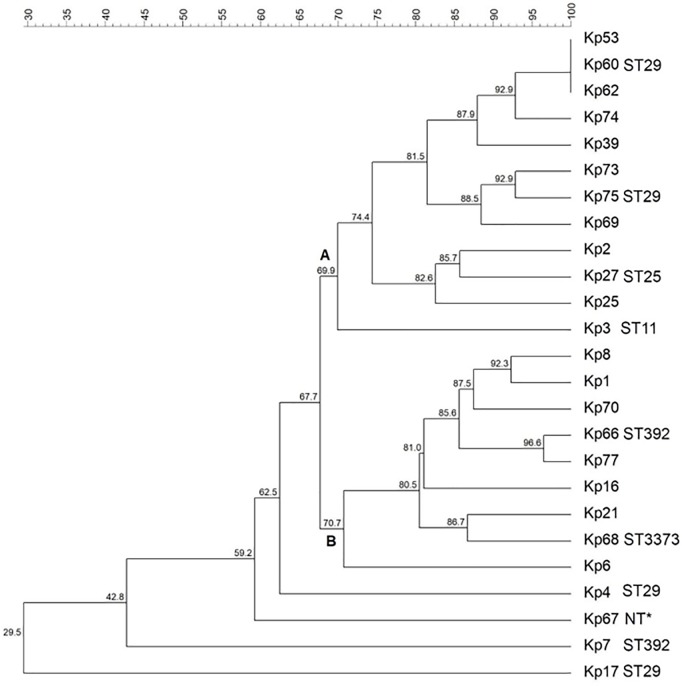
Dendrogram representing the genetic relationship among the 25 *Klebsiella pneumoniae* studied. Clusters were determined using the Unweighted Pair Group Mean (UPGMA) method and the Dice similarity coefficient. Similarity (%) among patterns is represented by the numbers beside the nodes. For each isolate typed by MLST, their respective sequence types (STs) are represented. ^∗^NT, not typed by MLST.

Genetic similarity among isolates was evaluated via ERIC-PCR, and the results indicated the vast majority of the isolates presented a rate of genetic similarity above 70%, separated into two main clusters (A and B) (Figure 1). Three isolates (Kp53, Kp60, and Kp62) showed 100% genetic similarity. Only four isolates (Kp4, Kp7, Kp17, and Kp67) were genetically more distant and did not cluster with the other isolates.

### MLST

Multilocus sequence typing analysis demonstrated five different STs among 10 selected isolates (Figure 1). Four isolates (Kp4, Kp17, Kp60, and Kp65) belonged to ST29, which was the most predominant group. Furthermore, two isolates (Kp7 and Kp66) belonged to ST392, one isolate (Kp27) belonged to ST25, and another one (Kp3) belonged to ST11. The isolate Kp68 presented a novel ST by way of a new allele combination, which was named ST3373. It was not possible to analyze the isolate Kp67 by MLST because it did not show amplification for the *ton*B gene, even after several attempts and adjustments in the reaction.

**FIGURE 2:**
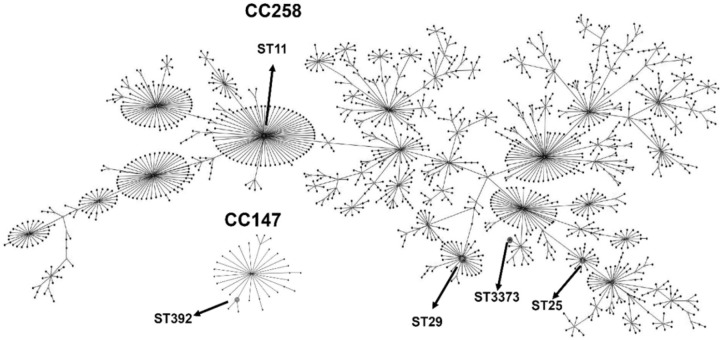
eBURST diagram generated with the MLST data, representing the five different sequence types (STs) obtained in this study (indicated by arrows), distributed in two clonal complexes: CC258, with the STs 11, 29, 25 and the novel ST3373 and CC147, with the ST392. The remaining STs were omitted from the diagram to facilitate visualization. Each dot represents an ST.

The eBurst analysis showed that most of the STs (STs 11, 25, 29, and 3373) found were distributed in a more massive clonal complex called CC258 (also called CC258/11). Only the ST392 group, including isolates Kp7 and Kp66, was present into a smaller clonal complex, called CC147 (Figure 2).

## Discussion

Although *K. pneumoniae* is considered to be an important opportunistic pathogen and a frequent cause of hospital-acquired infections (Struve and Krogfelt, 2004), it is also found in non-clinical habitats, which include the mucosal surfaces of humans and animals, and environmental sources such as water, soil, sewage, and vegetation (Bagley, 1985; Podschun et al., 2001). Previous studies have shown that *K. pneumoniae* strains of environmental origin are similar to those strains of clinical origin in terms of biochemical patterns, virulence, and pathogenicity (Podschun et al., 2001; Struve and Krogfelt, 2004); however, clinical *K. pneumoniae* are significantly more resistant to antibiotics as compared with environmental *K. pneumoniae* (Matsen et al., 1974).

In our study, the vast majority (84%, 21/25) of *K. pneumoniae* isolates showed MDR patterns including a high resistance rate to the common antibiotics used either alone or in association with one another to treat *K. pneumoniae* infections, such as β-lactams (including carbapenems), aminoglycosides, quinolones, glycylcycline, and polymyxin E. Although the high prevalence of MDR *K. pneumoniae* patterns was similar to other results in previous studies (Pereira et al., 2013; Paneru, 2015; Wasfi et al., 2016), this is the first report of a high incidence of MDR *K. pneumoniae* in the state of Tocantins, Brazil. There are many possible contributing factors to the emergence, rise, and spread of antibiotic resistance, including the new acquisition of resistance genes; transfer of antibiotic resistance genes; healthcare exposure; use of indwelling medical devices; limited diagnostic facilities; lack of effective and reliable surveillance systems; immunosuppressed states; travel to areas with a high endemicity of MDR bacteria; lack of new antimicrobial therapeutics; and inappropriate and excessive antibiotic use in health care, food-producing animals, and agriculture (Fletcher, 2015; Vila, 2015; Ayukekbong et al., 2017; Martin and Bachman, 2018; Patolia et al., 2018). Therefore, many of these risk factors may have contributed to the high rates of antibiotic resistance found in our study.

The high rates of resistance to polymyxin E (i.e., CST) and glycylcycline (i.e., TGC) found in our study deserves particular attention because these antibiotic categories have typically been used as the drugs of last resort for the treatment of severe infections caused by *Klebsiella pneumoniae* carbapenemase (KPC)-producing organisms (Pereira et al., 2013). Previous studies have reported that high levels of CST are frequently administered in Brazilian ICUs, mainly after bacteria isolates have become resistant to almost all other available antibiotics (Furtado et al., 2007; Rossi, 2011). Therefore, the overuse and misuse of antibiotics can be associated with an increase of the occurrence of CST resistance found in the current study. The TGC resistance might be due to the presence of the *Acr*AB gene, which encodes the efflux pump *Acr*AB and is considered to be one of the main contributors to a reduced susceptibility to TGC in *K. pneumoniae* clinical isolates (Bialek-Davenet et al., 2015; Wang et al., 2015; Elgendy et al., 2018). In this study, we also found that several TGC-resistant bacteria were isolated from rectal swabs, showing an important association between pathogen-specific and local antibiotic resistance patterns.

*K. pneumoniae* produces two classics trimeric porins, *Omp*K35 and *Omp*K36, which allow the passage of small hydrophilic molecules such as iron, nutrients, and antibiotics through the outer cell membrane (Tsai et al., 2011). In our study, 28% of all *K. pneumoniae* isolates lacked the *Omp*K36 gene. Our findings are in agreement with those of other authors who reported that the absence of *Omp*K35 or *Omp*K36 can be responsible for resistance to carbapenems in *K. pneumoniae* that produced ESBL (Hernandez-Alles et al., 1999; Wang et al., 2009; Skurnik et al., 2010). The loss of both porins *Omp*K35 and *Omp*K36 produces an increase in carbapenem, CIP, and chloramphenicol resistance (Kaczmarek et al., 2006). However, some of our results are not in complete agreement with the literature, as the presence of *Omp*K35 and *Omp*K36 genes were correlated with both carbapenem and CIP resistance, in 28% of MDR *K. pneumoniae* isolates. In contrast, other studies have suggested that the presence of both porins (*Omp*K35 and *Omp*K36) in MDR isolates can be associated with the presence of point mutations, disruption in the protein coding sequence, or promoter region mutations (Doumith et al., 2009; Wasfi et al., 2016). Further investigations should be performed to evaluate the presence of the mutations in bacteria strains isolated in this study.

Efflux pump systems have been reported as essential mechanisms of resistance and cause of MDR in *K. pneumoniae* (Mahamoud et al., 2007; Meletis et al., 2012). In *K. pneumoniae*, the *Acr*AB and *mdtK* complexes are the best-characterized efflux pumps (Wasfi et al., 2016). Notably, in our research, the presence of *Acr*AB-*Tol*C and *mdt*K genes were strongly associated with MDR *K. pneumoniae* patterns. These results are consistent with other previous studies, that demonstrated that the multidrug efflux pump system (*Acr*AB*-Tol*C) in *K. pneumoniae* was responsible for resistance to quinolones, tetracyclines, TGC, and beta-lactams in various MDR isolates (Padilla et al., 2010; Yuhan et al., 2016).

In *K. pneumoniae*, the genes *fim*H and *mrk*D encode adhesins of type 1 and type 3 fimbriae, which mediate binding to the extracellular matrix; promote biofilm development (Hornick et al., 1992; Struve et al., 2008; Alcántar-Curiel et al., 2013; Fu et al., 2018); and may play a key role in colonization, invasion and pathogenicity (Shah et al., 2017). In the current study, the majority of the MRD *K. pneumoniae* isolates carried both *fimH*-1 and *mrk*D virulence genes. Although studies have reported that many clinical *K. pneumoniae* isolates normally express both type 1 and type 3 fimbrial adhesins (Sahly et al., 2008; Struve et al., 2009; Wasfi et al., 2016), one of the most important steps in the progression to *K. pneumoniae* infection is related to its ability to adhere to host surfaces and demonstrate persistent colonization. *Mrk*D specifically mediates binding to the extracellular matrix, facilitating the adherence of *K. pneumoniae* to damaged tissue and coating indwelling devices (François et al., 1998; Paczosa and Mecsas, 2016), such as urinary catheters (Schroll et al., 2010; Stahlhut et al., 2012) and endotracheal tubes (François et al., 1998). Type 3 fimbriae were found to play an essential role in *K. pneumoniae* biofilm formation (Langstraat et al., 2001; Di Martino et al., 2003; Jagnow and Clegg, 2003; Schroll et al., 2010) and they can also mediate the binding of *K. pneumoniae* to endothelial cells and to epithelial cells of the respiratory and urinary tracts (Würker et al., 1990; Hornick et al., 1992; Tarkkanen et al., 1997). Type 1 fimbriae are expressed in 90% of both clinical and environmental *K. pneumoniae* isolates (Stahlhut et al., 2009); however, their precise role in the production of biofilms remains unclear (Paczosa and Mecsas, 2016). Type 1 fimbriae expressed by *K. pneumoniae* in particular cause urinary tract infections (Struve et al., 2008), and may play an important role in colonization of the intestine and in the delivery, entry, and persistence of *K. pneumoniae* in ventilator-associated pneumonia (Kollef, 2004; Struve et al., 2008; Kalanuria et al., 2014). Additionally, the presence of *mrk*D and *fim*H-1 has previously been associated with KPC-positive *K. pneumoniae* (De Cássia et al., 2014), which is in accordance with our findings. Although little is known regarding the potential virulence characteristics of KPC-producing *K. pneumonia* (Andrade et al., 2014; Liu Y. et al., 2014), studies have reported that ESBL-producing isolates of *K. pneumoniae* are able to produce more fimbrial adhesins, are more invasive, and are more resistant to the normal human serum bactericidal effect (Sahly et al., 2004). Therefore, the high frequency of *fim*H-1 (88%) and *mrk*D gene (96%) found in our results, illustrates the importance of evaluating these virulence factors.

The capsule is one of the most important virulence factors (Martin and Bachman, 2018) that protects *K. pneumoniae* from lethal serum factors and phagocytosis (Hsu et al., 2011). In *K pneumoniae*, capsular serotypes K1 and K2 have been considered as predominant virulent strains (Fung et al., 2002; Chuang et al., 2006). Studies using clinical samples have proposed that virulence factors such as K1, K2, K5, *rmp*A and the aerobactin gene, are absent in KPC-producing isolates (Siu et al., 2012). In agreement with these previous studies, our results showed that K1 and *rmp*A were not detected, K2 was present in only one isolate, K5 was not investigated, and all isolates were identified as KPC-producing *K. pneumoniae*. It is important to note that genes encoding *rmp*A, K1, or K2 were highly associated with the hypervirulent (hypermucoviscous) variant of *K. pneumoniae* (hvKP) (Fang et al., 2004; Yeh et al., 2007; Arena et al., 2017; Martin and Bachman, 2018), which causes serious community-acquired infection, and has emerged as a carbapenem-resistant hypervirulent *K. pneumoniae* (CR-HvKP) that can be found in clinical settings (Shon et al., 2013; Liu Y.M. et al., 2014; Zhang et al., 2015; Zhang Y. et al., 2016; Zhang R. et al., 2016). Therefore, this observation suggests that the *K. pneumoniae* in this study did not present molecular characteristics of the hypervirulent (hypermucoviscous) *K. pneumoniae*.

Siderophores are high-affinity, iron-chelating molecules that are critical for bacterial growth, replication, and virulence (Lawlor et al., 2007; Bachman et al., 2015; Holden and Bachman, 2015). The repertoire of siderophores differs among different strains (Behnsen and Raffatellu, 2016); thus, the role of each siderophore in virulence potential can vary (Paczosa and Mecsas, 2016; Lam et al., 2018). Siderophore-associated genes, such as *ent*B, *ybt*S and *iut*A are widely disseminated among *K. pneumonia* strains (Compain et al., 2014). However, *ent*B is only characterized for virulence when it occurs in association with *iut*A, *ybt*S, or *kfu* (Daehre et al., 2018). In agreement with previous studies, all *K. pneumoniae* carried the *ent*B gene (Lavigne et al., 2013; Fu et al., 2018); however, the presence of the genes encoding *ent*B in combination with *iut*A and *ybt*S was found in only 40%, while *ent*B with *ybt*S were found in 60% of all the strains, respectively. Although *K. pneumoniae* secretes a specific combination of siderophores, which can affect tissue localization, systemic spreading, and host survival, the effect of these molecules on the host during infection is not clear (Holden et al., 2016).

Carbapenems are the antibiotic class of choice for the treatment of severe infections caused by *Enterobacteriaceae*-producing ESBLs (Jacoby and Munoz-Price, 2005). The primary determinant of carbapenem resistance in *K. pneumoniae* is KPC-type carbapenemases (Nordmann et al., 2011), which are encoded by the gene *bla*_KPC_ and located mainly on a Tn3-based transposon, Tn4401 (Bina et al., 2015), demonstrating exceptional potential to spread throughout the world. In our findings, the presence of *bla*_KPC_ in all *K. pneumoniae* isolates is in agreement with previous investigations, that suggest the wide dissemination of KPC-producing isolates in various regions of Brazil (Castanheira et al., 2012; Pereira et al., 2013; Biberg et al., 2015; Gonçalves et al., 2017). Besides, PCR analysis demonstrated that most bacteria (84%) coproduced the *bla*_KPC_ and *bla*_OXA-1_ group resistance genes. In Brazil, several studies have reported the co-occurrence of *bla*_KPC_ with the *bla*_OXA-1_ group in *K. pneumoniae* (Fehlberg et al., 2012; Flores et al., 2016). Furthermore, *bla*_IMP_, *bla*_VIM_, *bla*_OXA48_, and *bla*_NDM_ are also genes that produce carbapenemases in *K. pneumoniae* (Lascols et al., 2012; Seibert et al., 2014); however, these genes were not found in our study.

Some reports have suggested that TEM (Temoniera), SHV (sulfhydryl variable), and CTX-M (cefotaxime-beta lactamases) are the primary genetic groups of ESBLs among clinically critical Gram-negative bacteria (Bradford, 2001; Paterson and Bonomo, 2005). Additional studies have indicated the presence of *bla*_CTX-M_, *bla*_TEM_, and *bla*_SHV_ genes in *K. pneumoniae* (Monteiro et al., 2009; Peirano et al., 2009; Seki et al., 2011; Fehlberg et al., 2012), which is in accordance with our results. Globally, the CTX-M type has appeared as the most common type of ESBL, and its incidence is easily surpassing those of SHV and TEM ESBLs in most locales (Jorgensen et al., 2010; Bora et al., 2014). Although our PCR analysis revealed that *bla*_TEM_ (100%) was the most frequent gene, followed by *bla*_SHV_ (96%), the presence of the *bla*_CTX-M_ (72%) group was also high, and can be related to the fluoroquinolone and aminoglycoside resistance (Pitout et al., 2005) found in this study. The co-production of *bla*_KPC_ with *bla*_TEM_ was detected in all isolates, while *bla*_KPC_, *bl*a_OXA_, *bla*_TEM_, *bla*_SHV_, and *bla*_CTX-M_ were observed in 72% and *bla*_KPC_, *bla*_TEM_, *bla*_SHV_, and *bla*_CTX-M_ were found in 68% of the *K. pneumoniae* isolates, respectively. Our results suggest that the high antimicrobial resistance found in this study can also be associated with the presence of these β-lactams genes.

Our ERIC-PCR results indicated that, although bacteria were isolated from different patients, the circulating *K. pneumoniae* in this hospital have a high genetic relationship to each other. Ten isolates belonging to the main ERIC-PCR clusters were analyzed by MLST, and four of them (Kp4, Kp17, Kp60, and Kp65) belonged to ST29. ST29 has previously been reported in *K. pneumoniae* strains from various parts of the world, such as Europe, Asia, Oceania, and also in Brazil. Uz Zaman et al. (1994) found ST29 in MDR *K. pneumoniae* carrying the OXA-48 gene that showed variations in outer membrane protein 36, causing an outbreak in a tertiary care hospital in Saudi Arabia. However, the isolates from our study with ST29 were negative for OmpK36 and OXA-48 (Tables 4, 5). The ST25 has been described as being associated with virulent clones, especially belonging to the capsular serotypes K1 and K2 (McCulloh and Opal, 2018). In our study, the only isolate that presented the K2 antigen (Kp27) and various virulence genes also presented the ST25; thus, our findings corroborate with the prior research (Table 5). ST11, found in the isolate Kp3, has been described as widespread in Brazil and is considered an international high-risk clone (Gonçalves et al., 2017).

eBURST analysis showed that, except for ST392, all other STs belong to the large clonal complex CC258. Commonly, *K. pneumoniae* isolates grouped into CC258 are associated with the production of carbapenemases and harbor many virulence genes (Gonçalves et al., 2017), which corroborates with our results (Table 5). Moreover, the ST392, found in the Kp66 isolate, is part of CC147, which is a small internationally successful clonal complex and has been shown to be an important epidemic clone. Hasan et al. (2014) described a clonal expansion of CC147 by Verone integron-encoded metallo-beta-lactamase (VIM)-producing *K. pneumoniae* strains isolated from Greece. ST392 has been reported worldwide as an emergent clone associated with the spreading KPC-producing *K. pneumoniae* (Yang et al., 2013; Di Mento et al., 2018; Garza-Ramos et al., 2018). In Brazil, ST392 was previously reported in a KPC-2-producing *K. pneumoniae* harboring the *mcr-*1 gene.

## Conclusion

Our results revealed a worrying situation concerning *K. pneumoniae* that is resistant to the drugs commonly used to treat infections and as well as those used as a last resort for life-threatening infections in patients admitted to the ICU. Additionally, our findings demonstrated the presence of high-risk international clones among isolates. Therefore, our data should be interpreted as an alert for need for prevention and control of the MDR *K. pneumoniae* in hospital settings. A careful and continued surveillance system that provides epidemiological and molecular information is important to limit the risk of infection and the spread of these strains.

## Author Contributions

RF, BS, and GR performed the experiments. MB kindly provided the strains and aided with the phenotypic detection of antibiotic resistance. ES aided with the writing and edition of the manuscript. EC aided with the sequencing analysis and the sequence submission to the NCBI platform. MCP, AC, AP-S, and CF conceived the idea, wrote the manuscript and analyzed the data. MLST and ERIC-PCR were performed by RN-S.

## Conflict of Interest Statement

The authors declare that the research was conducted in the absence of any commercial or financial relationships that could be construed as a potential conflict of interest.

## Footnotes

1 http://www.eucast.org/clinical_breakpoints/

2 https://blast.ncbi.nlm.nih.gov/

3 http://www.pasteur.fr/recherche/genopole/PF8/mlst/Kpneumoniae.html
