# Supplementary figures and images for: High Prevalence of Multidrug-Resistant Klebsiella pneumoniae Harboring Several Virulence and β-Lactamase Encoding Genes in a Brazilian Intensive Care Unit
Source: Front Microbiol. 2019 Jan 22;9:3198. doi: 10.3389/fmicb.2018.03198 (PMC6349766; doi:10.3389/fmicb.2018.03198)

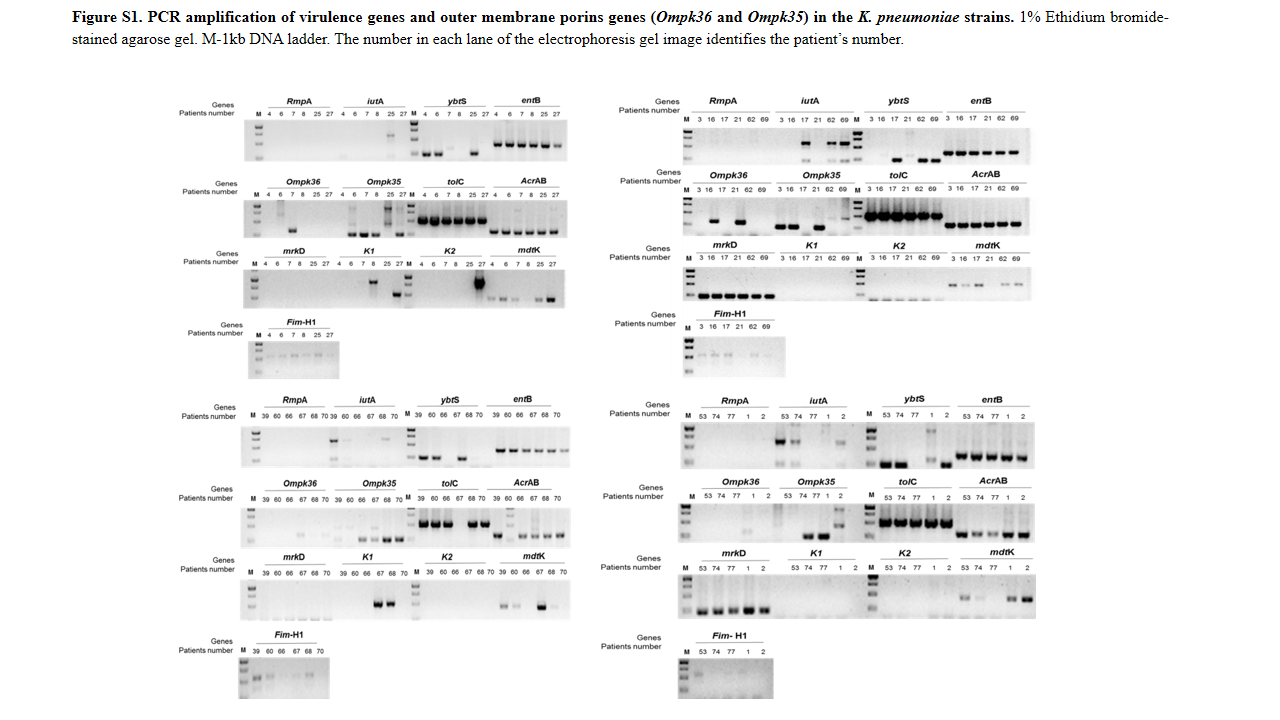

Supplement: Supplementary file 1 [file Image_1.TIF]

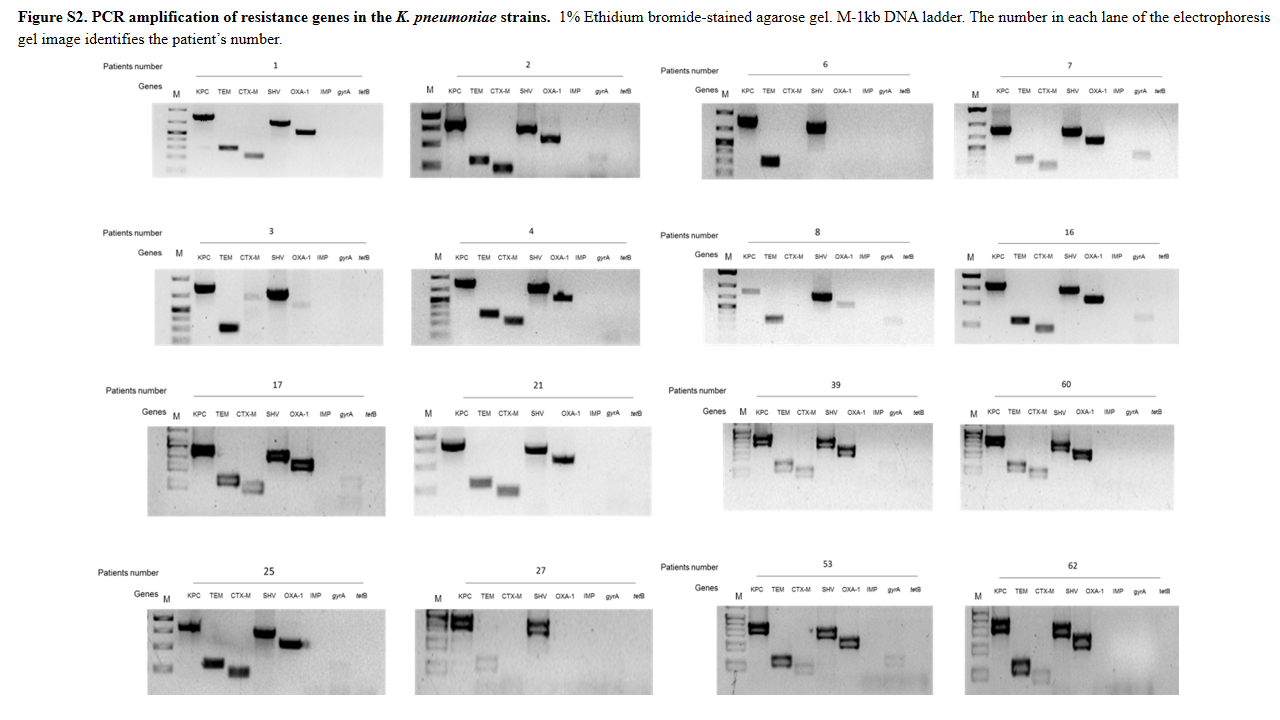

Supplement: Supplementary file 2 [file Image_2.TIF]

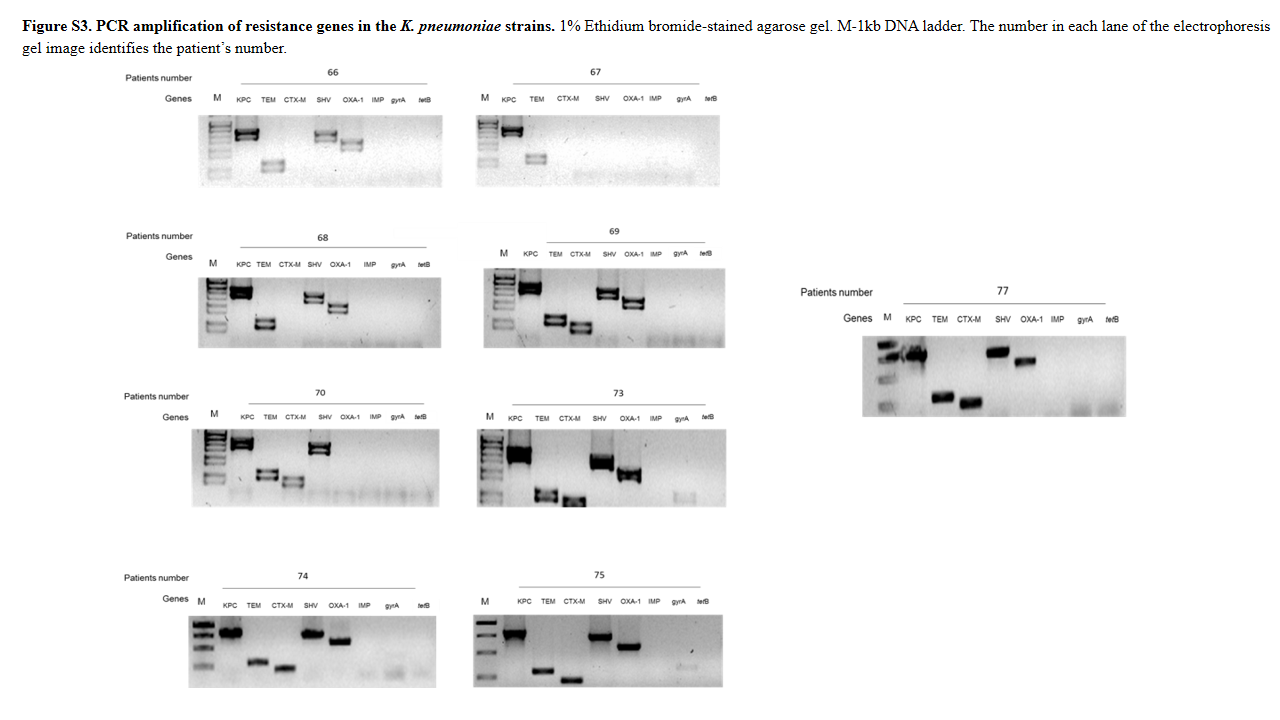

Supplement: Supplementary file 3 [file Image_3.TIF]
