# Supplementary material for: High Prevalence of Multidrug-Resistant Klebsiella pneumoniae Harboring Several Virulence and β-Lactamase Encoding Genes in a Brazilian Intensive Care Unit
Source: Front Microbiol. 2019 Jan 22;9:3198. doi: 10.3389/fmicb.2018.03198 (PMC6349766; doi:10.3389/fmicb.2018.03198)
Supplement: Supplementary file 4 [file Table_1.DOCX]

**Table S1. A**ntimicrobial profiles distributed by sex, age and tissue

| **Antibiotic resistance/Sex** | **Male** | | **Female** | | **p-value** |  |  |  |  |
| --- | --- | --- | --- | --- | --- | --- | --- | --- | --- |
|  | **Resistance** | **Sensitivity** | **Resistance** | **Sensitivity** |  |  |  |  |  |
| Amikacin | 1 | 13 | 0 | 11 | 1 |  |  |  |  |
| Gentamicin | 11 | 3 | 9 | 2 | 1 |  |  |  |  |
| Ciprofloxacin | 10 | 4 | 6 | 5 | 0,4341 |  |  |  |  |
| Tigecycline | 8 | 6 | 5 | 6 | 0,6951 |  |  |  |  |
| Colistin | 6 | 8 | 3 | 8 | 0,6766 |  |  |  |  |
|  |  | |  | |  |  |  |  |  |
|  |  |  |  |  |  |  |  |  |  |
| **Antibiotic resistance/Ages (years)** | **0-18 (1)** | | **19-59 (2)** | | **>60 (3)** | | **p-value 1/2** | **p-value 1/3** | **p-value 2/3** |
|  | **Resistance** | **Sensitivity** | **Resistance** | **Sensitivity** | **Resistance** | **Sensitivity** |  |  |  |
| Amikacin | 0 | 7 | 1 | 8 | 0 | 9 | 1 | 1 | 1 |
| Gentamicin | 5 | 2 | 8 | 1 | 7 | 2 | 0,55 | 1 | 1 |
| Ciprofloxacin | 6 | 1 | 4 | 5 | 6 | 3 | 0,1451 | 0,5846 | 0,6372 |
| Tigecycline | 5 | 2 | 4 | 5 | 4 | 5 | 0,3575 | 0,3575 | 1 |
| Colistin | 1 | 6 | 6 | 4 | 2 | 7 | 0,134 | 1 | 0,1698 |
|  |  |  |  |  |  |  |  |  |  |
|  |  |  |  |  |  |  |  |  |  |
| **Antibiotic resistance/Tissue** | **Rectal swab** | | **Tracheal aspirate** | |  |  |  |  |  |
|  | **Resistance** | **Sensitivity** | **Resistance** | **Sensitivity** | **p-value** |  |  |  |  |
| Amikacin | 1 | 13 | 0 | 4 | 1 |  |  |  |  |
| Gentamicin | 11 | 3 | 3 | 1 | 1 |  |  |  |  |
| Ciprofloxacin | 11 | 3 | 2 | 2 | 0,5327 |  |  |  |  |
| Tigecycline * | 11 | 3 | 0 | 4 | 0,0114 |  |  |  |  |
| Colistin | 5 | 9 | 0 | 4 | 0,2778 |  |  |  |  |

*p-value <0,05.
